# The predictive value of patient-reported outcomes on the impact of breast cancer treatment-related quality of life

**DOI:** 10.3389/fonc.2022.925534

**Published:** 2022-10-14

**Authors:** Ke Zhou, Martine Bellanger, Sophie Le Lann, Marie Robert, Jean-Sebastien Frenel, Mario Campone

**Affiliations:** ^1^ Department of Human and Social Sciences, Institut de Cancérologie de l’Ouest René Gauducheau, Saint-Herblain, France; ^2^ UMR CNRS6051 Rennes1 – EHESP School of Public Health, Rennes, France; ^3^ Department of Quality, Risk Management and Organization, Institut de Cancérologie de l’Ouest René Gauducheau, Saint-Herblain, France; ^4^ Department of Medical Oncology, Institut de Cancérologie de l’Ouest, René Gauducheau, Saint-Herblain, France; ^5^ CRCINA Team 8, UMR 1232 INSERM, Université de Nantes, Université d’Angers, Institut de Recherche en Santé-Université de Nantes, Nantes, France; ^6^ SIRIC ILIAD, Institut de Recherche en Santé-Université de Nantes, Nantes, France

**Keywords:** patient-reported outcomes, quality of life, breast cancer, chemotherapy, radiotherapy, targeted therapy, breast cancer surgery, value-based cancer care

## Abstract

**Purpose:**

Patient-reported outcomes (PROs) have been widely used to measure breast cancer (BC) treatment outcomes. However, evidence is still limited on using routinely PROs to personalize treatment decision-making, including or not chemotherapy, targeted therapy, and radiotherapy. Using patient baseline PRO scores, we aimed to use PROs before treatment initiation to predict improvement or decline in health-related quality of life (HRQoL) due to treatment that they receive.

**Methods:**

In two French cancer sites, women with non-metastatic BC completed the EORTC QLQ-C30 and QLQ-BR23 and BREAST-Q questionnaires to assess their PROs at baseline and again at 6 months. The outcome measured was post-operative change in PROs with minimal important difference for QLQ-C30 domains. We performed multivariate ordinal logistic regression to estimate the incremental probability of post-operative PRO improvements and deteriorations depending upon treatment options and baseline HRQoL.

**Results:**

One hundred twenty-seven women completed questionnaires. Chemotherapy had significant negative impacts on Global health status (GHS) and on physical and social functioning. Chemotherapy and radiotherapy increased patient fatigue scores after adjusting for clinical factors (p< 0.01 and p< 0.05, respectively). The incremental probability of GHS deteriorations for chemotherapy was +0.3, +0.5, and +0.34 for patients with baseline GHS scores of 40, 70, and 100, respectively. This showed that different pre-treatment PROs might predict differential effects of chemotherapy on women change in HRQoL.

**Conclusion:**

Patients with different baseline PRO scores may experience dissimilar impacts from BC treatments on post-operative PROs in terms of improvements and deteriorations. Oncologists might decide to adapt the treatment option based on a given level of the negative impact. Future studies should concentrate on incorporating this information into routine clinical decision-making strategies to optimize the treatment benefit for patients.

## Introduction

Collecting patient-reported outcomes (PROs), and their measures referred as PROMs, in breast cancer (BC) care has become of growing interest in most OECD (Organization for Economic Cooperation and Development) countries. PROMs are health status assessments that come directly from patients [e.g., functional status, symptoms that they experience, or quality of life (QoL) associated with their health condition and treatment] (1, 2).

PROs can complement clinical outcomes of BC treatments, mainly overall survival (OS) and disease-free survival (1, 3). Because survival rates of early BC have continuously improved over the last decades and are similar across care settings (4), addressing PROs alongside clinical outcomes could facilitate informed decision-making and enhance patient treatment by adapting care pathways. For example, the Patient-Reported Outcomes Measurement Information System^®^ (PROMIS^®^) monitors PROs after the treatment protocol starts. It reports significant treatment-induced side effects for clinicians to decide whether to discontinue treatments (5, 6).

In routine clinical practice, the choice of BC treatment and outcomes depends on factors such as hormone receptor status and the human epidermal growth factor receptor 2 (HER2) expression to predict recurrence risk (7). Despite their prognostic values successfully proven in oncology clinical trials (8), pre-treatment PROs have been barely used for trial stratification, as the recent study by Modi et al. shows (9). In addition, PROs remain underused routinely to determine individualized treatment protocols for BC prior to treatment initiation (7, 8), whereas combining PRO and clinical measures might enhance prediction of potential benefits and harms of BC treatments (9, 10).

Initiatives that the OECD has led, since 2017, such as “Measuring what matters - the patient reported indicator survey (PaRIS)” (11), have helped compare data on outcomes that matter for women with BC, from local providers within or between countries (12, 13). Compared with other European (EU) countries [such as The Netherlands (2) or Sweden (14)] and non-EU countries [such as the UK (1)], where health care organizations and the government have developed PROM programs, France has yet to implement PROMs in clinical practice in cancer settings. Our comprehensive cancer center, *Institut de Cancérologie de l’Ouest* (ICO) has been a pioneer by conducting in its two sites, a pilot study to collect PROs in patients with BC, as part of their 5-year strategy (2018–2022), with the prospect of a value-based care approach.

In this paper, we propose the use of real-world data of women with newly diagnosed non-metastatic BC to address the predictive value of PROs in daily clinical settings. We investigated the extent to which PROs measured before treatment initiation predict the effects of therapies on patients reported beneficial or detrimental outcomes. By using these pre-treatment PROs, we estimated the incremental probability of PRO improvements and deteriorations 6 months after surgery due to treatments that they received.

## Materials and methods

### Study overview

The study was carried out within the European project All.Can “Improving value in cancer care” in partnership with the International Consortium of Health Outcomes Measurement (ICHOM) (15). The project included 12 EU hospitals or cancer centers. Analysis was based on clinical outcomes along with PROs collected at the ICO using the *Breast Cancer Data Collection Reference Guide* published by the ICHOM (15).

### Study population

Patients were recruited over a 6-month period between 13 December 2018 and 23 May 2019 in ICO Saint-Herblain and Angers sites. Eligible patients were aged ≥18 years and with first primary diagnosis of invasive non-metastatic BC or ductal carcinoma *in situ*. Treatment approaches including surgery [breast conserving therapy (BCT) and mastectomy (Mx)], (neo) adjuvant chemotherapy (CT) ± targeted therapy (TT) (i.e., Trastuzumab), radiotherapy (RT), and hormone therapy (HT) are all performed at the ICO cancer center sites. Patients with rare tumors, lobular carcinoma *in situ*, or recurrent disease were excluded. When eligibility criteria are met, clinicians offered patients the opportunity to participate in the study during their initial consultation. All patients signed a written consent form to participate in the study that the Ethics Committee (Angers University Hospital 2019/14) approved on 19 March 2019.

### PROMs

PROMs included in the set were the EORTC QLQ-C30 (16), the EORTC-QLQ Breast Cancer (BR23) (17), and BREAST-Q (18). For the latter, ICO outcomes were previously published in the OECD “Health at a glance” 2019 and 2021 (4, 13).

Participants completed paper- or web-based questionnaires at baseline: before the first treatment (T0), following neo-adjuvant CT (T3), and 6 months after surgery (T6). Data collection time points were based on the European project All.Can timeline. However, for consistency in the data collected and ICO treatment pathway, our analysis used the PRO measures at two time points: “baseline and post-operative”, i.e., T0 and T6.

The EORTC QLQ-C30 includes 30 items within the global health status (GHS)/QoL domain, five functional domains (i.e., physical, role, emotional, cognitive, and social), and nine symptom domains such as fatigue, pain, and insomnia. We obtained the scores of each domain according to the method from the EORTC scoring manual (19). The BC-specific module, EORTC QLQ-BR23, consists of four functional domains (i.e., body image, sexual functioning, sexual enjoyment, and future perspective) and four symptom domains (i.e., systemic therapy side effects, breast symptoms, arm symptoms, and upset by hair loss). All scores are based on a scale of 0 to 100, with higher scores representing better health-related QoL (HRQoL) for functional domains, and inverse relationship for symptom domains, based on standardized protocol for validating the questionnaires used in the study (19).

### Statistical analysis

We described patients’ characteristics by reporting median and interquartile range (IQR) for age and body mass index (BMI), as well as the number and percentage of patients for categorical variables.

The change in PRO scores (CS), i.e., change in patients’ self-reported health status after surgery, was calculated as post-operative scores minus baseline scores. We interpreted CS with minimal important differences (MIDs) following the guidelines by Cocks et al. (20). We estimated the MID for the QLQ-C30 questionnaire only and not for the QLQ-BR23, as done in previous studies (21, 22). To our knowledge, no MID estimate has been determined for this module. We defined patient categories using CS as an ordinal variable with three levels: “deteriorations”, “no change”, and “improvements” in PRO scores. We identified patients with ceiling scores (i.e., having 100) in baseline measurements and conducted sensitivity analysis accordingly. When relevant, we tested interaction between treatment modalities. Because of a high questionnaire response rate, we did not perform any imputation of missing data. We described the BR23 results as a complement of the QLQ-C30 analysis.

In univariate analysis, we identified patient characteristics related to deteriorations and improvements in all QLQ-C30 domains.

On a swimmer plot, we described patients’ treatment modalities and the post-operative PRO changes. We plotted therapies in chronological order on a timeline and used bars to show CS in global health status. In addition, we produced bar plots to represent population level post-operative changes in all other PRO domains.

We performed multivariate ordinal logistic regression of deteriorations and improvements in PROs after receiving BC therapies. The dependent variables were GHS, physical and emotional functioning, as well as fatigue and pain outcomes, whereas baseline scores in these same domains, binary variables of the above therapies (i.e., with or without that therapy), BC subtype, BC stage, and age at diagnosis were adjustment variables. To estimate the impact of treatment, we compared the predicted probabilities of PRO deterioration and improvements for “a therapy” versus “no therapy” (23). The estimated difference between these predicted probabilities was referred as incremental probability and defined (24, 25) as follows:


(1) Incremental probability=Pr(Event=1|Trt=1)−Pr(Event=1|Trt=0)

where Event can be either PRO improvements or deteriorations, and Trt can be one among CT, RT, HT, and TT.

When we estimated the incremental probability, we fixed one baseline PRO score at once and repeated the process for all possible scores. All other variables in the model remained at their original values (i.e., all other variables being equal).

Last, we made visual comparisons between therapies by plotting baseline PRO scores (x-axis) versus incremental probabilities of PRO “outcomes” (y-axis).

The DxCare^®^ medical information system was used for data collection. All statistical analyses were performed with STATA^®^ 14.2 package.

## Results

### Baseline characteristics

Of the 179 women recruited over 6 months, 127 (71%) completed QLQ-C30 questionnaire.

Overall, 10 (8%) of the respondents had stage 0 BC (*in situ*), 99 (78%) had early stage BC (stages I to IIA), and 18 (14%) had locally advanced BC (stages IIB to III). Invasive BC subtypes were divided as follows: luminal BC (n = 87; 69%), basal-like BC (n = 14; 11%), and HER2+ (n = 17; 13%). One hundred ten (87%) women underwent BCT and 17 (13%) Mx. RT (n = 118; 93%) was the most received adjuvant treatment followed by HT (n = 97; 76%). More patients received adjuvant CT (n = 31; 24%) than neo-adjuvant CT (n = 19; 15%) (**Table 1**).

**Table 1:** Patient characteristics (N = 127).

Patient characteristics	n (%)
Age at diagnosis, median (IQR)	62 (52–71)
Age under 50 years	24 (19)
Age above 50 years	103 (81)
Menopausal status	
Pre-menopausal	35 (28)
Post-menopausal	92 (72)
Laterality	
Left	58 (46)
Right	66 (52)
Bilateral	2 (2)
BMI, median (IQR)	24 (22–29)
Obesity (BMI > 25)	
No	64 (50)
Yes	63 (50)
Number of comorbidities ^1^	
0	71 (56)
1	37 (29)
>2	19 (15)
Cancer stage ^2^	
0 (*In situ*)	10 (8)
Invasive	
I	68 (54)
IIA	31 (24)
IIB	13 (10)
IIIA	3 (2)
IIIB	2 (2)
Breast cancer subtype (Invasive cancer only) ^3^	
Luminal	87 (69)
Basal-like	14 (11)
Her2+	17 (13)
Chemotherapy ^4^	
No	77 (61)
Yes	50 (39)
Targeted therapy ^4^	
No	113 (89)
Yes	14 (11)
Radiotherapy	
No	9 (7)
Yes	118 (93)
Hormone therapy ^5^	
No	30 (24)
Yes	97 (76)

^1^Multiple comorbidities: The total number of comorbidities including heart disease, high blood pressure, leg pain, lung disease, diabetes, kidney disease, liver disease, stroke sequelae, nervous system diseases, other cancers, depression, and arthritis.

^2^Cancer stages: According to UICC TNM classification eighth edition, 2017.

^3^Breast cancer subtype: Determined by estrogen receptor status (ER), progesterone receptor status (PR), human epidermal growth factor receptor 2 status (HER2); “Luminal” includes any positive status of ER and PR, plus HER2-negative status (HR+, HER2−); “Basal-like”, also called “triple negative” means EP-, PR-, and HER2-negative status; “Her2+” including all other status. “Missing” includes patients had any unperformed test of the receptors.

^4^Chemotherapy (CT) and targeted therapy (TT): Epirubicin and cyclophosphamide followed by Paclitaxel or Docetaxel, associated with Trastuzumab or not; according to personalized treatment protocol; 19 patients received neo-adjuvant chemotherapy, and 31 received adjuvant chemotherapy. Duration of undergoing CT before post-operative questionnaire: Median 3.8 months, IQR (3.5–4.4), mean 3.8, and range (0.7–5).

^5^Hormone therapy (HT): Anastrozole, Letrozole, and Tamoxifen; according to personalized treatment protocol. Duration of undergoing HT before post-operative questionnaire: Median 2.3 months, IQR (1.9–3), mean 0.57, and range (0.3–8.5); 33 patients received both HT and CT; and 17 and 64 patients received CT or HT alone, respectively.

### Improvements and deteriorations in EORTC QLQ-C30 scores

**Figure 1:**
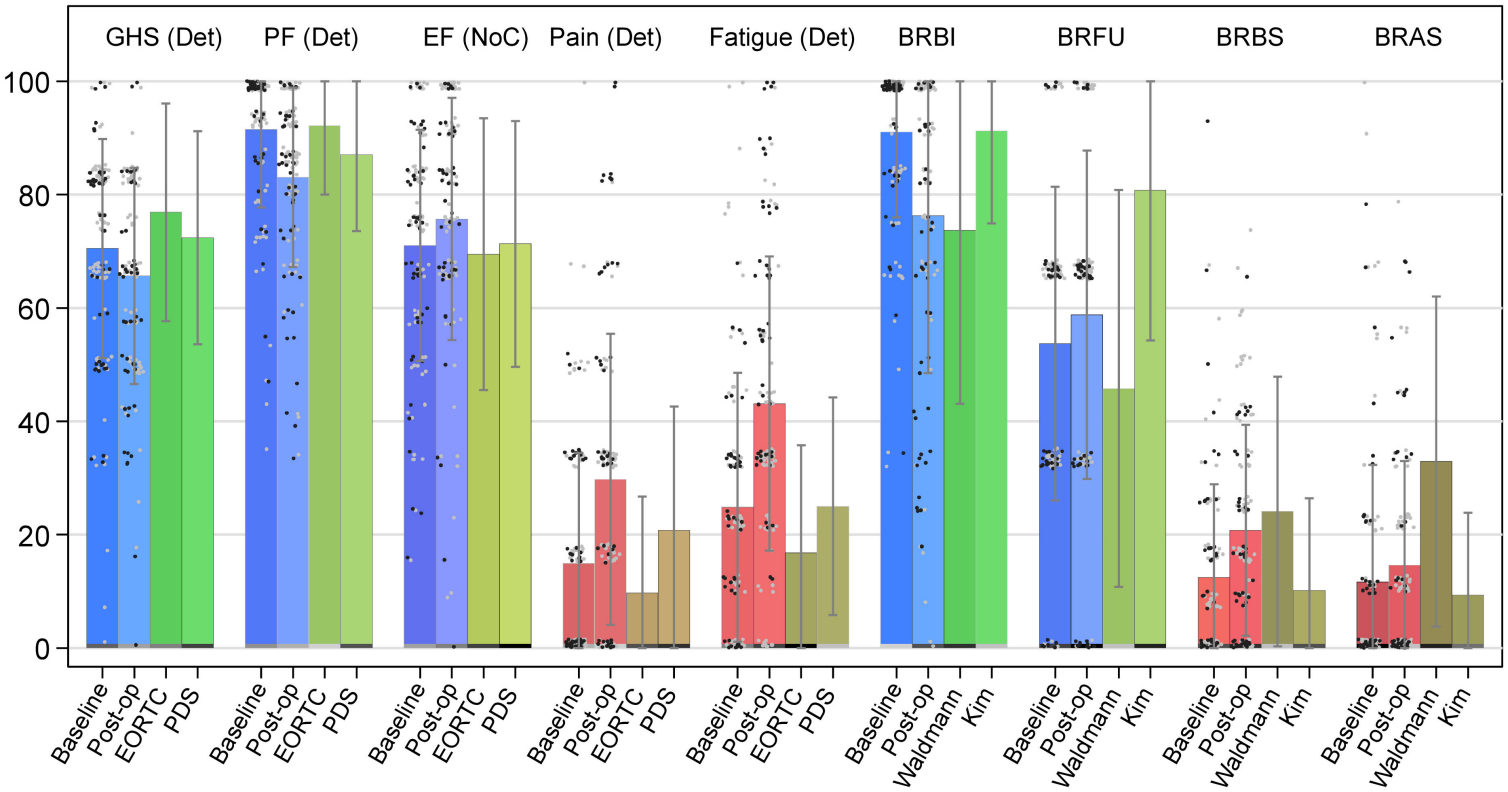
Bar chart of selected QLQ-C30 and QLQ-BR23 domain scores. Notes: ICO ICHOM data compared with reference values from Mierzynska et al. (2020) (EORTC and PDS) (26) and from Waldmann et al. (2007) and Kim et al. (2012); Error bars, standard deviation (SD); gray dots, patients without chemotherapy; black dots, patients with chemotherapy; GHS, global health status/QoL domain; PF, physical functioning domain; EF, emotional functioning domain; Pain, pain symptom domain; Fatigue, fatigue symptom domain; BRBI, body image; BRFU, future perspective; BRBS, breast symptoms; BRAS, arm symptoms; GHS, PF, EF, BRBI, and BRFU: Higher scores represent better health/HRQoL; Pain, Fatigue, BRBS, and BRAS: Higher scores represent more symptoms/poorer HRQoL. Of note, for QLQ-C30, Det, Imp, and NoC, Deteriorations, Improvements, and No Change indicate whether population mean score change reached minimal important difference (MID) and significance level of p-value<0.05 with the Wilcoxon signed-rank test for paired data. Baseline: ICO ICHOM data, baseline scores, measured at initial consultation for the announcement of surgery. Post-op: ICO ICHOM data, baseline scores, measured 6 months after the baseline measure. EORTC (27): Closed RCTs data identified from the European Organization for Research and Treatment of Cancer (EORTC) database. PDS (28): Project Data Sphere databases, an independent non-profit platform designed to provide patient-level data from RCTs. Waldmann (29): Data from the study published by Waldmann and colleagues (2007). Kim (30): Data from the study published by Kim and colleagues (2012).

On average, the study population experienced all functioning scores worsening except of the emotional (**Figure 1**). We observed symptom deteriorations for fatigue, pain, dyspnea, and constipation. Interestingly, no domain reached MID improvement in terms of population mean.

The 6-month post-operative PROMs showed that more than half of the women reported GHS improvements or no change (**Table 2**; **Figure 1**). Large proportions of deteriorations were noted in physical and role functioning scores (64% and 51%, respectively), whereas emotional functioning showed more improvements than deteriorations (50% versus 32%). Very high HRQoL was reported before the intervention by a majority of women, when considering baseline social and role functioning scales that had a ceiling effect of 86% and 72%, respectively, and by more than half of them for physical and cognitive functioning (**Supplementary Figure 1**). Unexpectedly, 64% (n = 51) of the women who had worse post-operative HRQoL in physical functioning (n = 80) had the maximum score at baseline. Regarding symptoms, HRQoL was worse, for fatigue, pain, and, to a lesser extent, dyspnea (**Table 2**). Only appetite loss (AP) had similar proportions of deteriorations and improvements in scores.

**Table 2:** Patients with deteriorations, improvements, and no change in QLQ-C30 domain scores, defined by minimal important difference (MID) thresholds provided by Cocks et al. (2011) (20).

QLQ-C30 domains	MID groups		
	Deteriorations	No Change	Improvements
	n (%)	n (%)	n (%)
Function			
Global Health Status (N = 124)	56 (45)	36 (29)	32 (26)
Physical Functioning (N = 125)	80 (64)	29 (23)	16 (13)
Role functioning (N = 124)	63 (51)	51 (41)	10 (8)
Emotional Functioning (N = 125)	40 (32)	24 (19)	61 (49)
Cognitive functioning (N = 126)	51 (40)	56 (44)	19 (15)
Social functioning (N = 125)	57 (46)	60 (48)	8 (6)
Symptom			
Fatigue (N = 125)	83 (66)	20 (16)	22 (18)
Nausea and Vomiting (N = 126)	23 (18)	94 (75)	9 (7)
Pain (N = 126)	63 (50)	49 (39)	14 (11)
Dyspnea (N = 123)	49 (40)	63 (51)	11 (9)
Insomnia (N = 125)	37 (30)	56 (45)	32 (26)
Appetite loss (N = 125)	22 (18)	82 (66)	21 (17)
Constipation (N = 124)	30 (24)	81 (65)	13 (10)
Diarrhoea (N = 124)	26 (21)	85 (69)	13 (10)
Financial difficulties (N = 121)	26 (21)	88 (73)	7 (6)

**Figure 2:**
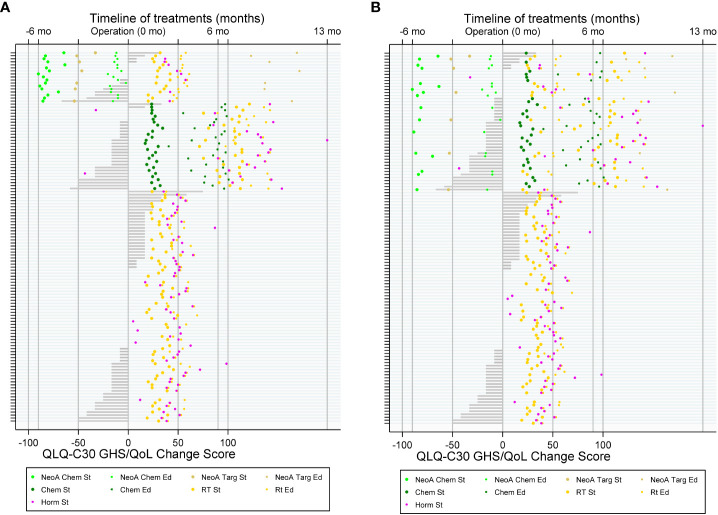
Treatment flow of patients with breast cancer (BC) and patient-reported outcome (PRO) with QLQ-C30 Global Health Status (GHS) change score. **(A)** By three groups: with neo-adjuvant chemotherapy (top), with adjuvant chemotherapy (middle), and without chemotherapy (bottom); **(B)** by two groups: with and without chemotherapy.

Univariate analysis showed that, in women with CT versus no CT, higher proportions had a worsened GHS and a lower proportion of improved GHS (p< 0.01) (**Figures 1**, **2**). We observed similar findings in physical and emotional functioning (p< 0.01) as well as in social functioning (p< 0.001) (**Supplementary Table 1**). Negative impacts on fatigue were proportionally higher in women with RT vs. no RT and CT vs. no CT (p< 0.001). Women above 50 years compared with younger women enjoyed higher proportions in physical functioning improvements. However, women under 50 years were more likely to have a ceiling effect compared to their older counterparts (88% versus 49%, p< 0.001). Obviously, a patient having a ceiling effect at baseline can have either no change or lower scores in later follow-ups. Women with luminal BC versus those with other BC subtypes had better HRQoL for physical (p< 0.05) and for role functioning (p< 0.01).

### Multivariate analysis and incremental probability of PRO improvements and deteriorations due to BC therapies

**Figure 3:**
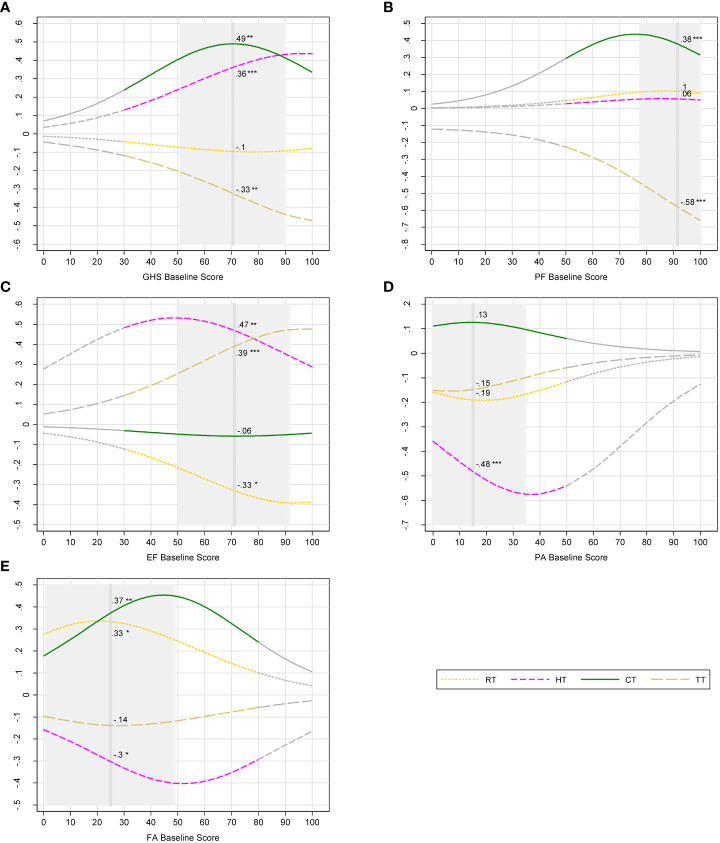
Incremental probability of post-operative outcomes of breast cancer therapies. **(A)** Global health status deteriorations; **(B)** physical functioning deteriorations; **(C)** emotional functioning improvements; **(D)** pain deteriorations; **(E)** fatigue deteriorations. Notes: Breast cancer therapies: RT, radiotherapy; HT, hormone therapy; CT, chemotherapy; TT, targeted therapy. Post-operative outcomes were measured with European Organization for the Research and Treatment of Cancer Quality of Life Questionnaire (EORTC QLQ-C30). QLQ-C30 domains: GHS, global health status; PF, physical functioning; EF, emotional functioning; PA, pain; FA, fatigue. Higher scores represent better health-related quality of life (HRQoL) for GHS, PF, and EF. In the contrary, higher scores represent more severe problems and lower HRQoL for PA and FA. Deteriorations and improvements where defined with minimal important difference (MID) of change score (post-operative score minus baseline score). Gray part of curves: zones in which the number of participants is less than 5; vertical dash line: mean baseline score; Gray area ± 1 SD from mean baseline score, truncated if it crosses the minimum score (0) or the maximum score (100). Incremental probabilities were derived from ordinal logistic regression. The incremental probability indicates the differential probability of deteriorations/improvements in PROs brought by one therapy. For each baseline score, the incremental probability is equal to the probability of deteriorations/improvements with one therapy minus the probability without the therapy. Numbers beside the curves indicate the incremental probability at mean baseline scores. Significance level: null hypothesis = the incremental probability is equal to zero at mean baseline score; *p-value< 0.05, **< 0.01, and ***< 0.001. Example: Receiving CT increases significantly the probability of GHS deteriorations by 49% given that a patient had a baseline GHS score of 71.

Impacts of different BC therapies from multivariate analysis are shown in **Figure 3**. CT had significant negative impacts on GHS and on physical and social functioning. CT and RT increased patient fatigue scores (p< 0.01 and p< 0.05, respectively) (**Supplementary Table 2**). When women had baseline GHS score of 71 (i.e., equivalent to that of the population mean), receiving CT (versus no CT) significantly changed the probability of 6-month GHS deteriorations by +0.5 (**Figure 3A**) and by about +0.4 that of physical functioning and fatigue deteriorations, respectively (**Figures 3B, E**). We observed for RT (versus no RT) a decrease in the incremental probability of improvements in emotional functioning (**Figure 3C**) and a likely increase of having more severe fatigue at mean operative scores (**Figure 3A**). Women undergoing TT (versus no TT) were less likely to have deterioration in GHS and physical activity (−0.3 and −0.6, respectively) (**Figures 3A, B**) and more likely to improve emotional functioning (+0.4) (**Figure 3C**).

Overall, the predicted incremental probabilities vary depending on baseline scores. The incremental probability of GHS deteriorations for CT was +0.3, +0.5, and +0.34 for patients with baseline GHS scores of 40, 70, and 100, respectively (**Figure 3A**). Patients with different PRO baseline scores did not report experience the same level of harms associated with CT.

Finally, sensitivity analysis showed that having a ceiling effect at baseline did not change our results. We found significant interactions between treatment options. The deterioration effect of receiving CT and RT on GHS and fatigue was amplified when the two treatments were combined.

### EORTC QLQ-BR23 scores

As shown in **Figure 1**, 64% of women had a ceiling score for body image at baseline. The mean score of that same domain decreased by 15 points after surgery (p< 0.001). The mean score of future perspective increased by five points (p< 0.01). It is worth noting that this domain and the emotional functioning (QLQ-C30) domain have improved after surgery (p< 0.05).

## Discussion

In this paper, we explored the change in PRO scores from baseline to 6 months after surgery among women with newly diagnosed non-metastatic BC. By modeling real-world data, we investigated the likelihood of specific BC treatment options to improve or deteriorate PROs based on baseline scores.

Estimates showed that CT significantly influenced PROs in a negative direction for several HRQoL domains, consistently with previous findings (31, 32). RT led to more severe fatigue and negative impacts on emotional functioning as well. Our analysis demonstrated a synergistic effect on GHS and fatigue deterioration when patients receive CT and RT together, in line with previous studies (33, 34). These findings held true after adjusting for BC subtype, BC stage, and age at diagnosis.

Our study demonstrates that pre-treatment PROs predict benefits and harms of BC therapies in terms of 6-month PRO improvements and deteriorations. Baseline PRO scores enabled us to differentiate therapeutic impacts between patients. Clinically, our findings on GHS deteriorations because of CT reflect that, for patients with poor self-reported health scores at baseline (GHS = 40), deteriorations due to CT were relatively less severe compared with those with average self-reported health scores at baseline (GHS = 70). Patients with good self-reported health scores at baseline (GHS = 100) might tolerate CT side effects better and therefore had less severe health deteriorations reported. Patients’ baseline self-reported health status could predict the extent by which CT would have detrimental effects on women with BC undergoing such treatment. This can help develop the “use of patient reported questionnaires that incorporate HRQoL measures as well as clinically interpreted measures” (9) to decide whether to use a treatment.

The question remains: “When is the incremental probability of deterioration in QoL critical enough for readapting a treatment option?” Clinicians should offer clinically relevant thresholds to adjust treatment options. As an example, clinicians and patients can compare their interpretation of score level using the bookmark approach as suggested by Rorthrock et al. in PROMIS^®^ measures of physical function and cognitive function in patients with cancer. This will help develop and disseminate guidelines that will be useful for different cancer types and in different settings (5).

Interestingly, in our analysis, BC stage, subtype, and age at diagnosis had only limited impacts on PROs despite their important clinical prognosis value at 6 months. The higher probability of physical functioning improvements among older patients was associated to a large proportion of younger patients having ceiling effects.

Furthermore, previous studies found “the prognostic significance of baseline PROs as independent predictors of the overall survival of patients with cancer in clinical studies.” (8, 35) To some extent, our results confirmed such findings. This enables clinicians, informed by patients’ pre-treatment PROs, to make personalized treatment plans and integrate PROs into the whole cycle of care, i.e., from diagnosis to post-treatment surveillance (10).

We learnt from this pilot study that some barriers persist to reach desirable features of a routinely implemented PROMs system. This concerns patients, health professionals, and IT systems. Understandable questionnaires help patients reporting their outcomes with reasonable time and efforts. Clearly defined administrative protocols enable the integration of PROMs into clinicians’ workflow without additional workload. A secured information system providing clinically interpretable results and alerts of critical symptoms is vital to an efficient use of PROMs (36–38), as this is envisioned in our cancer center.

A shift toward “PROs that reflect value” (39) would be possible as suggested by the value-based health care (VBHC) approach (40), as already implemented in several OECD countries (3, 36). VBHC aims to improve quality of care and provider performance by evaluating structure and care pathways using PROMs (15). This requires that PROs should be incorporated into the clinical decision-making process (41). Toxicity monitoring along with PROs significantly optimizes response to supportive care needs (42–44) and consequently leads to better OS (45) and HRQoL (37).

The main limitation of our study is that we collected data for HRQoL over a limited time due to the constraint of timeline set, whereas the effects of a long-term treatment such as HT could be thoroughly measured with a longer data collection period. However, patients with BC had the most significant PRO deteriorations at 6 months after surgery (32, 36, 46). This underlines the rebound effect, which is observed in patients with BC who experience immediate post-diagnosis drop of PROMs, after which, improvements take place progressively (47). Our results are consistent with previous studies that found that PRO scores decreased between baseline (T0) and 6 months post-operatively (T6) followed by improvements in physical or psychological symptoms that we could observe with a longer data collection period (32, 36, 46).

Our study has notable strengths. This was the first study using real-world data from two French cancer center sites measuring PROs of different treatment options of BC. This study will help introducing PROMs into routine data collection in cancer centers. Our study opens the perspective of linking pre-treatment HRQoL/PRO scores with clinical indicators, such as OS as a composite outcome (PRO × OS) in personalized treatment of BC, because OS and disease progression are crucial clinical outcomes to consider prior to patient HRQoL.

## Conclusion

Patients with different baseline PRO scores may experience different impacts from BC treatments on post-operative PROs in terms of improvements and deteriorations. Future studies are to confirm these findings. The confirmation will lead to the incorporation of PROs/PROMs into routine clinical decision-making strategies to optimize patients’ HRQoL following BC treatment.

## Data availability statement

The raw data supporting the conclusions of this article will be made available by the authors, without undue reservation.

## Author contributions

KZ: conceptualization, methodology, software programming, formal analysis, manuscript drafting and revision, and visualization. MB: conceptualization, methodology, manuscript drafting, revision and validation, and supervision. SL: investigation and data curation. MC, MR, and J-SF: manuscript validation. All authors contributed to the article and approved the submitted version.

## Funding

This work received financial support from the Institut de Cancérologie de l’Ouest René Gauducheau, to cover the publication costs.

## Acknowledgments

We thank Dr. Stacey Johnson and Sandy Laham for their helpful comments.

## Conflict of interest

The authors declare that the research was conducted in the absence of any commercial or financial relationships that could be construed as a potential conflict of interest.

## Publisher’s note

All claims expressed in this article are solely those of the authors and do not necessarily represent those of their affiliated organizations, or those of the publisher, the editors and the reviewers. Any product that may be evaluated in this article, or claim that may be made by its manufacturer, is not guaranteed or endorsed by the publisher.

## References

[1] Porter I Gonçalves-Bradley D Ricci-Cabello I Gibbons C Gangannagaripalli J Fitzpatrick R. Framework and guidance for implementing patient-reported outcomes in clinical practice: Evidence, challenges and opportunities. J Comp Effectiveness Res (2016) 5:507–19. doi: 10.2217/cer-2015-0014 27427277

[2] van Egdom LSE Oemrawsingh A Verweij LM Lingsma HF Koppert LB Verhoef C. Implementing patient-reported outcome measures in clinical breast cancer care: A systematic review. Value Health (2019) 22:1197–226. doi: 10.1016/j.jval.2019.04.1927 31563263

[3] Lagendijk M Mittendorf E King TA Gibbons C Pusic A Dominici LS. Incorporating patient-reported outcome measures into breast surgical oncology: Advancing toward value-based care. Oncologist (2020) 25:384–90. doi: 10.1634/theoncologist.2019-0355 PMC7216450 31848315

[4] OECD. Health at a glance 2019: OECD indicators. Paris: OECD (2019). doi: 10.1787/4dd50c09-en

[5] Rothrock NE Cook KF O’Connor M Cella D Smith AW Yount SE. Establishing clinically-relevant terms and severity thresholds for patient-reported outcomes measurement information system^®^ (PROMIS^®^) measures of physical function, cognitive function, and sleep disturbance in people with cancer using standard setting. Qual Life Res (2019) 28:3355–62. doi: 10.1007/s11136-019-02261-2 PMC6868299 31410640

[6] Seneviratne MG Bozkurt S Patel MI Seto T Brooks JD Blayney DW. Distribution of global health measures from routinely collected PROMIS surveys in patients with breast cancer or prostate cancer. Cancer (2019) 125:943–51. doi: 10.1002/cncr.31895 PMC6403006 30512191

[7] Burguin A Diorio C Durocher F. Breast cancer treatments: Updates and new challenges. J Pers Med (2021) 11:808. doi: 10.3390/jpm11080808 34442452 PMC8399130

[8] Mierzynska J Piccinin C Pe M Martinelli F Gotay C Coens C. Prognostic value of patient-reported outcomes from international randomised clinical trials on cancer: A systematic review. Lancet Oncol (2019) 20:e685–e698. doi: 10.1016/S1470-2045(19)30656-4 31797795

[9] Modi ND Danell NO Perry RNA Abuhelwa AY Rathod A Badaoui S. Patient-reported outcomes predict survival and adverse events following anticancer treatment initiation in advanced HER2-positive breast cancer. ESMO Open (2022) 7:100475. doi: 10.1016/j.esmoop.2022.100475 35490579 PMC9271483

[10] Jordan K Aapro M Kaasa S Ripamonti CI Scotté F Strasser F. European Society for medical oncology (ESMO) position paper on supportive and palliative care. Ann Oncol (2018) 29:36–43. doi: 10.1093/annonc/mdx757 29253069

[11] OECD. Measuring what matters: The patient-reported indicator surveys. Paris, France: OECD (2019).

[12] OECD Breast Cancer Patient Reported Outcomes Working Group, Guidelines for International Data Collection. OECD working document. Organisation for Economic Co-operation and Development (2019). 1-7, OECD Paris. Available at: https://www.oecd.org/health/paris/patient-reportedindicatorsurveysparis-documents.htm [Accessed 30 March, 2022].

[13] OECD. Health at a glance 2021: OECD indicators. Paris: OECD (2021). doi: 10.1787/ae3016b9-en

[14] Eriksson M Anveden L Celebioglu F Dahlberg K Meldahl I Lagergren J. Radiotherapy in implant-based immediate breast reconstruction: Risk factors, surgical outcomes, and patient-reported outcome measures in a large Swedish multicenter cohort. Breast Cancer Res Treat (2013) 142:591–601. doi: 10.1007/s10549-013-2770-0 24258257

[15] Ong WL Schouwenburg MG van Bommel ACM Stowell C Allison KH Benn KE. A standard set of value-based patient-centered outcomes for breast cancer: The international consortium for health outcomes measurement (ICHOM) initiative. JAMA Oncol (2017) 3:677–85. doi: 10.1001/jamaoncol.2016.4851 28033439

[16] Aaronson NK Ahmedzai S Bergman B Bullinger M Cull A Duez NJ. The European organization for research and treatment of cancer QLQ-C30: A quality-of-Life instrument for use in international clinical trials in oncology. JNCI: J Natl Cancer Inst (1993) 85:365–76. doi: 10.1093/jnci/85.5.365 8433390

[17] Sprangers MA Groenvold M Arraras JI Franklin J te Velde A Muller M. The European organization for research and treatment of cancer breast cancer-specific quality-of-life questionnaire module: first results from a three-country field study. JCO (1996) 14:2756–68. doi: 10.1200/JCO.1996.14.10.2756 8874337

[18] Pusic AL Klassen AF Scott AM Klok JA Cordeiro PG Cano SJ. Development of a new patient-reported outcome measure for breast surgery: The BREAST-q. Plast Reconstr Surg (2009) 124:345–53. doi: 10.1097/PRS.0b013e3181aee807 19644246

[19] Fayers P Aaronson NK Bjordal K Sullivan M. EORTC QLQ–C30 scoring manual, in: European Organisation for research and treatment of cancer (1995). Available at: https://abdn.pure.elsevier.com/en/publications/eortc-qlqc30-scoring-manual-2 (Accessed January 5, 2022).

[20] Cocks K King MT Velikova G Martyn St-James M Fayers PM Brown JM. Evidence-based guidelines for determination of sample size and interpretation of the European organisation for the research and treatment of cancer quality of life questionnaire core 30. JCO (2011) 29:89–96. doi: 10.1200/JCO.2010.28.0107 21098316

[21] Karsten MM Roehle R Albers S Pross T Hage AM Weiler K. Real-world reference scores for EORTC QLQ-C30 and EORTC QLQ-BR23 in early breast cancer patients. Eur J Cancer (2022) 163:128–39. doi: 10.1016/j.ejca.2021.12.020 35066338

[22] Tripathy D Curteis T Hurvitz S Yardley D Franke F Babu KG. Correlation between work productivity loss and EORTC QLQ-C30 and -BR23 domains from the MONALEESA-7 trial of premenopausal women with HR+/HER2– advanced breast cancer. Ther Adv Med Oncol (2022) 14:175883592210812. doi: 10.1177/17588359221081203 PMC8891884 35251320

[23] Williams R. Using the margins command to estimate and interpret adjusted predictions and marginal effects. Stata J (2012) 12:308–31. doi: 10.1177/1536867X1201200209

[24] Morise AP Detrano R Bobbio M Diamond GA. Development and validation of a logistic regression-derived algorithm for estimating the incremental probability of coronary artery disease before and after exercise testing. J Am Coll Cardiol (1992) 20:1187–96. doi: 10.1016/0735-1097(92)90377-Y 1401621

[25] Zikmund-Fisher BJ. The right tool is what they need, not what we have: A taxonomy of appropriate levels of precision in patient risk communication. Med Care Res Rev (2013) 70:37S–49S. doi: 10.1177/1077558712458541 22955699

[26] Mierzynska J Taye M Pe M Coens C Martinelli F Pogoda K. Reference values for the EORTC QLQ-C30 in early and metastatic breast cancer. Eur J Cancer (2020) 125:69–82. doi: 10.1016/j.ejca.2019.10.031 31838407

[27] EORTC. EORTC clinical trial database. Available at: https://www.eortc.org/clinical-trials-database/ (Accessed January 10, 2022).

[28] PDS. Project data sphere. Available at: https://www.projectdatasphere.org/ (Accessed January 10, 2022).

[29] Waldmann A Pritzkuleit R Raspe H Katalinic A. The OVIS study: Health related quality of life measured by the EORTC QLQ-C30 and -BR23 in German female patients with breast cancer from schleswig-Holstein. Qual Life Res (2007) 16:767–76. doi: 10.1007/s11136-006-9161-5 17286196

[30] Kim E Ko S-K Kang H-Y. Mapping the cancer-specific EORTC QLQ-C30 and EORTC QLQ-BR23 to the generic EQ-5D in metastatic breast cancer patients. Qual Life Res (2012) 21:1193–203. doi: 10.1007/s11136-011-0037-y 22012023

[31] Battisti NML Reed MWR Herbert E Morgan JL Collins KA Ward SE. Bridging the age gap in breast cancer: Impact of chemotherapy on quality of life in older women with early breast cancer. Eur J Cancer (2021) 144:269–80. doi: 10.1016/j.ejca.2020.11.022 PMC7896040 33373871

[32] Ferreira AR Di Meglio A Pistilli B Gbenou AS El-Mouhebb M Dauchy S. Differential impact of endocrine therapy and chemotherapy on quality of life of breast cancer survivors: A prospective patient-reported outcomes analysis. Ann Oncol (2019) 30:1784–95. doi: 10.1093/annonc/mdz298 31591636

[33] Battisti NML Hatton MQ Reed MWR Herbert E Morgan JL Bradburn M. Observational cohort study in older women with early breast cancer: Use of radiation therapy and impact on health-related quality of life and mortality. Radiother Oncol (2021) 161:166–76. doi: 10.1016/j.radonc.2021.06.021 34146616

[34] Macquart-Moulin G Viens P Genre D Bouscary M-L Resbeut M Gravis G. Concomitant chemoradiotherapy for patients with nonmetastatic breast carcinoma. Cancer (1999) 85:2190–9. doi: 10.1002/(SICI)1097-0142(19990515)85:10<2190::AID-CNCR13>3.0.CO;2-P 10326697

[35] Efficace F Collins GS Cottone F Giesinger JM Sommer K Anota A. Patient-reported outcomes as independent prognostic factors for survival in oncology: Systematic review and meta-analysis. Value Health (2021) 24:250–67. doi: 10.1016/j.jval.2020.10.017 33518032

[36] van Egdom LSE Lagendijk M van der Kemp MH van Dam JH Mureau MAM Hazelzet JA. Implementation of value based breast cancer care. Eur J Surg Oncol (2019) 45:1163–70. doi: 10.1016/j.ejso.2019.01.007 30638807

[37] Di Maio M Basch E Bryce J Perrone F. Patient-reported outcomes in the evaluation of toxicity of anticancer treatments. Nat Rev Clin Oncol (2016) 13:319–25. doi: 10.1038/nrclinonc.2015.222 26787278

[38] Basch E Barbera L Kerrigan CL Velikova G. Implementation of patient-reported outcomes in routine medical care. Am Soc Clin Oncol Educ Book (2018) 38:122–34. doi: 10.1200/EDBK_200383 30231381

[39] Porter ME. What is value in health care. N Engl J Med (2010) 363:2477–81. doi: 10.1056/NEJMp1011024 21142528

[40] Porter ME Kaplan RS. How to pay for health care. Harvard Business Rev (2016) 94:88–98, 100, 134. 27526565

[41] Casali P Licitra L Constantini M Santoro A Viterbori P Bajetta E. Quality of life assessment and clinical decision-making. Ann Oncol (1997) 8:1207–11. doi: 10.1023/A:1008276901910 9496385

[42] Diplock BD McGarragle KMC Mueller WA Haddad S Ehrlich R Yoon D-HA. The impact of automated screening with Edmonton symptom assessment system (ESAS) on health-related quality of life, supportive care needs, and patient satisfaction with care in 268 ambulatory cancer patients. Support Care Cancer (2019) 27:209–18. doi: 10.1007/s00520-018-4304-0 29931490

[43] Garcia SF Wortman K Cella D Wagner LI Bass M Kircher S. Implementing electronic health record–integrated screening of patient-reported symptoms and supportive care needs in a comprehensive cancer center. Cancer (2019) 125:4059–68. doi: 10.1002/cncr.32172 PMC11345862 31373682

[44] Marthick M McGregor D Alison J Cheema B Dhillon H Shaw T. Supportive care interventions for people with cancer assisted by digital technology: Systematic review. J Med Internet Res (2021) 23:e24722. doi: 10.2196/24722 34714246 PMC8590193

[45] Basch E Deal AM Dueck AC Scher HI Kris MG Hudis C. Overall survival results of a trial assessing patient-reported outcomes for symptom monitoring during routine cancer treatment. JAMA (2017) 318:197–8. doi: 10.1001/jama.2017.7156 PMC5817466 28586821

[46] Lagendijk M van Egdom LSE Richel C van Leeuwen N Verhoef C Lingsma HF. Patient reported outcome measures in breast cancer patients. Eur J Surg Oncol (2018) 44:963–8. doi: 10.1016/j.ejso.2018.03.009 29678302

[47] Bourdon M Blanchin M Tessier P Campone M Quéreux G Dravet F. Changes in quality of life after a diagnosis of cancer: a 2-year study comparing breast cancer and melanoma patients. Qual Life Res (2016) 25:1969–79. doi: 10.1007/s11136-016-1244-3 26886927

