# Supplementary material for: The predictive value of patient-reported outcomes on the impact of breast cancer treatment-related quality of life
Source: Front Oncol. 2022 Oct 14;12:925534. doi: 10.3389/fonc.2022.925534 (PMC9613969; doi:10.3389/fonc.2022.925534)
Supplement: Supplementary file 1 [file Table_1.pdf]

# The Predictive Value of Patient-Reported Outcomes on the Impact of Breast Cancer Treatment-Related Quality of Life

## *Supplementary Material*

Supplementary Table 1. Univariate analysis. Determinants of clinically important deterioration and improvements of QLQ-C30 domain scores

| Patient characteristics | QLQ-C30 Global Status and Functions | | | | | | | | | | | | QLQ-C30 Symptoms and Bothers | | | | | | | | | | | | | | | | | |
|---|---|---|---|---|---|---|---|---|---|---|---|---|---|---|---|---|---|---|---|---|---|---|---|---|---|---|---|---|---|---|
| | GHS N=123 | | PF N=125 | | RF N=123 | | EF N=125 | | CF N=126 | | SF N=125 | | FA N=125 | | NV N=125 | | PA N=126 | | DY N=123 | | SL N=124 | | AP N=125 | | CO N=122 | | DI N=121 | | FI N=120 | |
| | ↘ | ↗ | ↘ | ↗ | ↘ | ↗ | ↘ | ↗ | ↘ | ↗ | ↘ | ↗ | ↘ | ↗ | ↘ | ↗ | ↘ | ↗ | ↘ | ↗ | ↘ | ↗ | ↘ | ↗ | ↘ | ↗ | ↘ | ↗ | ↘ | ↗ |
| Chemotherapy | ** | ** | ** | ** | ** | ** | | | | | *** | * | *** | ** | | | | | | | | | * | | | | * | | | |
| Radiotherapy | | | | | | | | | | | | | *** | | | | | | | | | | | | | ** | | | | |
| Hormone therapy | | | | | | | * | | | | | | | | | | | | | | | | | | | | | | | |
| Targeted therapy | | | | | | | | | | | | | | | | | | | * | | | ** | | | | | | | | |
| Age at diagnosis | | | | * | | | | | | | | | | | | | | | | | | | | | | | | | | |
| Marital status | | | | | | | | | | | | | | | | | | | | | | | | | | | | | | |
| Menopause stat. | | | | | * | | | | | | | | | | | | | | | | | * | | ** | | | | | | |
| Laterality | | | | | | | | | | | | | | | | | | | | | | | | | | | | | | |
| Obesity | | | | | | | | * | | | | | | | | | | | | | | | | | | | | | | |
| Histological typ. | | | | | | | | | | | | * | | | | | | | | | | | | | | | | | | |
| ER | | | | | | | | | | | | | | | | | | | | | | | | | | | | | | |
| PR | | | | | | | | | | | | | | | | | | | | | | | | | | | | | | |
| HER2 | | | | | | | | | | | | | | | | | | | | | | | | | | | | | | |
| HR+,HER2- | | | * | | ** | | | | | | | * | | | | | | | * | | | | | | | | | | | |
| Triple negative | | | | | | | | | | | | | | | | | | | | | | | | | | | | | | |
| N. of comorbidities | | | | | | | | * | | | | | | | | | | | | | | | * | | | | | | | |

Notes: QLQ-C30 domain outcomes: ↘ - Deteriorations ↗- Improvements. The association between patient characteristics and QLQ-C30 domain outcomes were tested using Fisher’s exact test; * p-value<.05 **<.01 ***<.001. Age at diagnosis included two categories divided by 50 years of age. GHS – Global Health Status / QoL domain; PF – Physical Functioning domain; RF – Role Functioning; EF – Emotional Functioning domain; CR – Cognitive Functioning; SF – Social Functioning; FA – Fatigue; NV – Nausea and Vomiting; PA – Pain ; DY – Dyspnoea; SL – Insomnia; AP – Appetite Loss; CO – Constipation; DI – Diarrhoea; FI – Financial Difficulties.
