# Supplementary material for: The predictive value of patient-reported outcomes on the impact of breast cancer treatment-related quality of life
Source: Front Oncol. 2022 Oct 14;12:925534. doi: 10.3389/fonc.2022.925534 (PMC9613969; doi:10.3389/fonc.2022.925534)
Supplement: Supplementary file 2 [file Table_2.pdf]

The Predictive Value of Patient-Reported Outcomes on the Impact of Breast Cancer Treatment-Related Quality of Life

Supplementary Material

Supplementary Table 2. Result summary of multivariate analysis, QLQ-C30 domain with significant (p-value<.05) postoperative impact of BC treatments

Chemotherapy	GHS +0.5(Det)	PF +0.4(Det)		SF +0.3(Det)	FA +0.4(Det)				
Radiotherapy			EF -0.3(Imp)		FA +0.3(Det)				
Hormone therapy	GHS +0.4(Det)		EF +0.5(Imp)		FA -0.3(Det)	NV +0.2(Det)	PA -0.5(Det)		
Targeted therapy	GHS -0.3(Det)	PF -0.6(Det)	EF +0.4(Imp)		RF -0.4(Det)	NV -0.2(Det)		SL -0.2(Det)	AP -0.2(Det)

Notes: GHS – Global Health Status / QoL domain; PF – Physical Functioning domain; EF – Emotional Functioning domain; FA – Fatigue; NV – Nausea and Vomiting; PA – Pain ; SL – Insomnia; AP – Appetite Loss; Det/Imp – Deteriorations/Improvements.
Green color: a BC treatment has positive postoperative impact on a domain with decreasing probabilities of having deteriorations or increasing probabilities of having improvements. Red color: a BC treatment has negative postoperative impact on a domain with increasing probabilities of having deteriorations or decreasing probabilities of having improvements.
The number indicates the differential probability of deteriorations/improvements in PROs brought by one therapy at mean baseline score. For each baseline score, the incremental probability equals the probability of deteriorations/improvements with one therapy minus the probability without the therapy.
Example: Receiving CT increases significantly the probability of GHS deteriorations by about 50% given that a patient had a mean baseline score.
