# Supplementary figures and images for: The predictive value of patient-reported outcomes on the impact of breast cancer treatment-related quality of life
Source: Front Oncol. 2022 Oct 14;12:925534. doi: 10.3389/fonc.2022.925534 (PMC9613969; doi:10.3389/fonc.2022.925534)

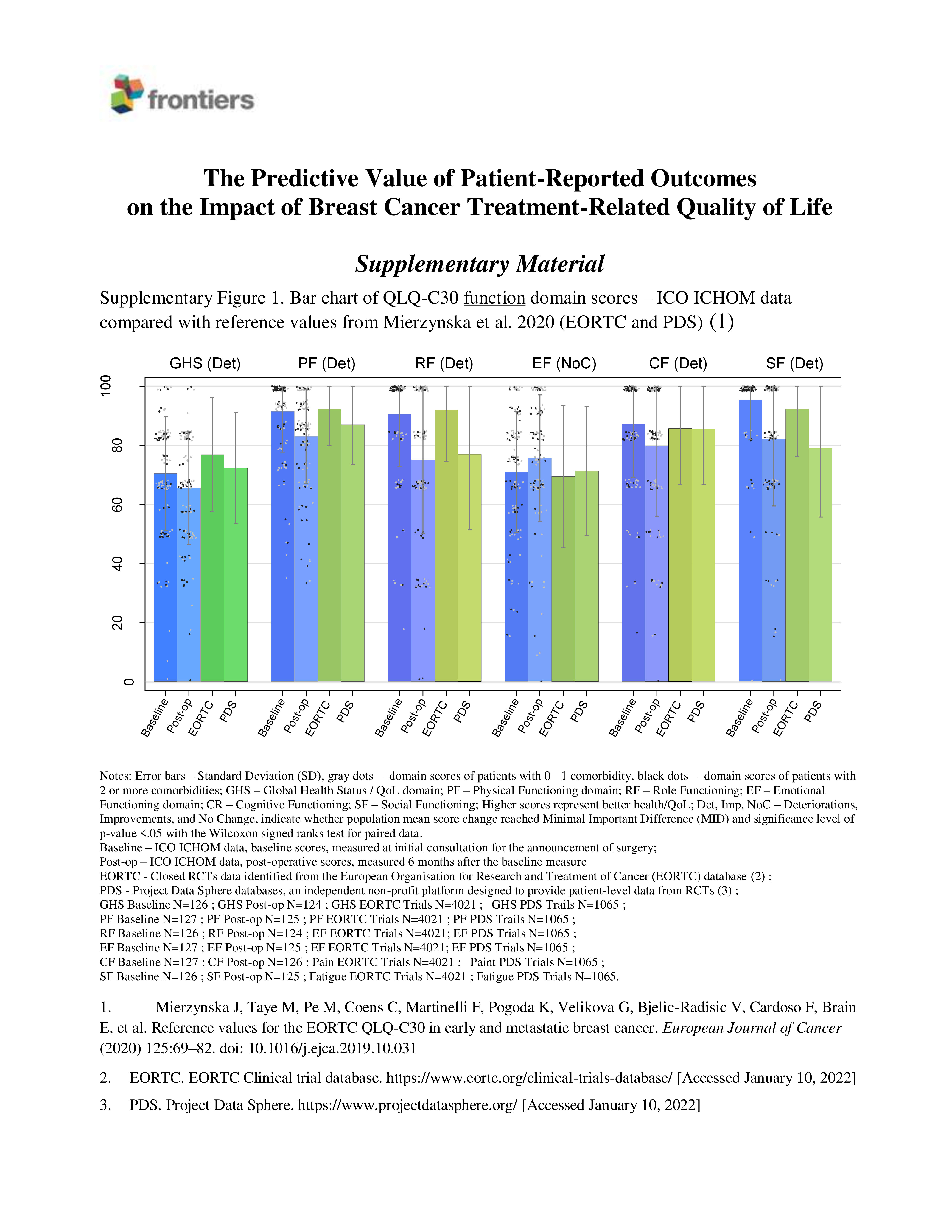

Supplement: Supplementary file 3 [file Image_1.tiff]
